# Neutrophil Extracellular Traps in Periodontitis

**DOI:** 10.3390/cells9061494

**Published:** 2020-06-19

**Authors:** Antonio Magán-Fernández, Sarmad Muayad Rasheed Al-Bakri, Francisco O’Valle, Cristina Benavides-Reyes, Francisco Abadía-Molina, Francisco Mesa

**Affiliations:** 1 Periodontology Department, School of Dentistry, University of Granada, 18071 Granada, Spain; amaganf@ugr.es (A.M.-F.); sarmad89@correo.ugr.es (S.M.R.A.-B.); fmesa@ugr.es (F.M.); 2 Pathology Department, School of Medicine (IBIMER, CIBM), University of Granada, 18071 Granada, Spain; fovalle@ugr.es; 3 Biosanitary Research Institute (IBS-GRANADA), University of Granada, 18012 Granada, Spain; 4 Department of Operative Dentistry, School of Dentistry, University of Granada, 18071 Granada, Spain; 5 Department of Cell Biology, University of Granada, 18071 Granada, Spain; fmolina@ugr.es; 6 INYTA, Institute of Nutrition and Food Technology “José Mataix”, University of Granada, Armilla, 18100 Granada, Spain

**Keywords:** innate immunity, periodontitis, neutrophil functions, neutrophil extracellular traps

## Abstract

Neutrophils are key cells of the immune system and have a decisive role in fighting foreign pathogens in infectious diseases. Neutrophil extracellular traps (NETs) consist of a mesh of DNA enclosing antimicrobial peptides and histones that are released into extracellular space following neutrophil response to a wide range of stimuli, such as pathogens, host-derived mediators and drugs. Neutrophils can remain functional after NET formation and are important for periodontal homeostasis. Periodontitis is an inflammatory multifactorial disease caused by a dysbiosis state between the gingival microbiome and the immune response of the host. The pathogenesis of periodontitis includes an immune-inflammatory component in which impaired NET formation and/or elimination can be involved, contributing to an exacerbated inflammatory reaction and to the destruction of gingival tissue. In this review, we summarize the current knowledge about the role of NETs in the pathogenesis of periodontitis.

## 1. Periodontitis

Periodontitis is a chronic inflammatory disease that affects the tooth-supporting tissues and exhibits a wide range of clinical, microbiological and immunological manifestations. It is associated with, and caused by, a multifaceted dynamic interaction among specific infectious agents, host immune responses, hazardous environmental exposure and genetic propensity [1]. The process of developing the disease starts with the accumulation of a complex bacterial biofilm. The composition of this biofilm has been estimated in approximately 700 species [2]. This biofilm creates a coat for the dental root and its structure is capable of protecting against antimicrobial substances. In healthy subjects, there is a homeostasis between the periodontium and the host response. However, when the plaque biofilm persists in a susceptible host it generates an inflammatory reaction that causes a dysbiosis, where periodontal pathogens thrive [3]. This leads to a chronic inflammatory state, which consequentially causes the destruction of the connective tissue. The process can progress to destroying surrounding support tissues—gingiva, cementum, periodontal ligament and alveolar bone—and eventually end in the loss of the affected teeth [4].

As a result of gingivitis, the bacteria penetrate the sulcus between the gum and the tooth, and then attack the gum attachment to progress deeper along the root. During this migration, toxins produced by bacteria and consequent inflammatory reactions will irreversibly destroy the attachment and the tooth-supporting tissues. This leads to the formation of periodontal pockets, which are located between the deep periodontal tissues and the tooth and are considered to be the main clinical feature of periodontitis [5].

The frequency and severity of periodontitis increases with age, with incidence peaking around the age of 60 [6]. Periodontitis is considered the main cause of tooth loss in people older than 40, having a higher prevalence than caries [7]. A high prevalence of periodontitis has been reported, with more than 47% of adults (more than 60 million) in the USA affected, and the prevalence continues to grow every year [8,9].

Periodontal inflammation is characterized by a chronic inflammatory infiltrate of varying intensity. This infiltrate is mainly composed of lymphocytes, plasma cells and macrophages distributed in patches on the lamina propria, frequently surrounding vascular structures [10]. Neutrophils are abundant in the periodontal inflammatory-immune response infiltrate and are considered a first-line cell defense mechanism against bacterial invasion [11]. However, in a susceptible host in which neutrophils do not properly contribute to the restraint of the invading bacteria, the homeostasis between the biofilm and the host response is altered, leading to an increase in tissue destruction [12,13]. Due to this immune-inflammatory component, periodontitis has been related to several systemic diseases, including rheumatoid arthritis (RA) [14]. Previous studies have indicated that neutrophils derived from patients with periodontitis are hyperactive and have an increased activity and production of reactive oxygen species (ROS) in response to a microbial invasion [15].

## 2. Periodontal Neutrophils

Neutrophils are the most abundant cell type of the granulocyte family (95%) and represent 50% to 70% of the blood leukocytes [11], approximately (1–2) × 10^11^ neutrophils are produced daily and released from the bone marrow into the bloodstream [16]. Peripheral blood neutrophils are eventually recruited from the bloodstream into the site of the infection. Naturally present in the oral cavity, neutrophils attach to the endothelial cells through the interaction with selectin and integrin receptors; by extravasation they abandon the bloodstream and migrate from the periodontal sulcus into the oral cavity. In case of infection, neutrophils are the first of the immune cells to arrive at the site through periodontal tissues and into gingival crevices as part of normal immune control. Although neutrophils are one of the predominant immune cells present in the oral cavity, T cells in periodontal tissue constitute the prevalent immune cell type [17]. Additionally, oral neutrophils have been found to show different chemotactic and antimicrobial functions than circulating neutrophils [18,19].

Mutations in genes affecting neutrophil differentiation and egression from the bone marrow have been related to periodontitis. Severe periodontitis has been described in patients with Severe Congenital Neutropenia due to mutations in the neutrophil elastase (NE) ELA2/ELANE or the HAX1 gene (hematopoietic cell-specific Lyn substrate) 1-associated gene X1 [20]. Patients with warts, hypogammaglobulinemia, immunodeficiency and myelokathexis (WHIM) syndrome have been reported to present with severe periodontitis [21,22]. WHIM is an autosomal dominant immunodeficiency caused by mutations in the CXCR4 chemokine receptor leading to defects in neutrophil exiting from the bone marrow.

Different neutrophil defects have been described affecting all stages of neutrophil recruitment and extravasation to periodontal tissue: tethering, rolling, adhesion and endothelial transmigration [23,24]. Most notably, leukocyte adhesion deficiency-I (LAD-I) immunodeficiency, which alters neutrophil extravasation into tissues, presents with periodontitis [25]. LAD-I results from mutation in the CD18 gene [26] preventing normal integrin dimerization and leukocyte adhesion and extravasation. Endothelial cell-derived developmental endothelial locus-1 (DEL-1) inhibits neutrophil adhesion to the endothelial cells [27] thereby restraining neutrophil transmigration; consequently, both DEL-1 upregulation and deficiency have been related to periodontitis [28].

Periodontitis is associated with reduced neutrophil chemotaxis. Dysfunctional neutrophil chemotaxis may predispose patients with periodontitis to disease by increasing tissue transit times, thereby exacerbating neutrophil-mediated collateral host tissue damage [29]. The absence of tissue neutrophils due to defective recruitment and extravasation [23] can also lead to persistent periodontal inflammation and bone loss [30,31]. Both an excessive presence or absence of neutrophils in the tissue can lead to periodontitis, indicating how important neutrophil balance is in periodontal homeostasis. A comprehensive understanding of defective neutrophil behavior in periodontitis would help in the development of new therapeutic approaches.

## 3. Neutrophil Extracellular Traps (NETs)

### 3.1. NET Formation

Neutrophils contribute to host defense at sites of tissue injury by patroling through the circulatory system [32]. The function of eliminating invading pathogens in periodontal tissues is mediated through ROS production, phagocytosis, extracellular and intracellular degranulation [11] and most recently neutrophil extracellular trap (NET) production. Brinkmann first described NETs as bactericidal traps, disarming and promoting the elimination of extracellular bacteria [33]. The formation of NETs involves the extrusion of nuclear chromatin into the extracellular space through the rupture of the nuclear and plasma membranes, and this extruded chromatin is embedded with cytoplasmic granule-derived proteins [34]. The term NETosis has been used in the past years to describe the combination of NET formation and neutrophil death [35]. However, concerns have been raised and the use of this term has been discouraged in some reports, and other terms such as “NET formation” or “NETotic cell death” have been proposed [36,37]. NETs are web-like structures of decondensed nuclear chromatin fibers combined with various antimicrobial compounds, including histones and antimicrobial peptides (AMPs) from azurophilic granules, specific granules and tertiary granules (gelatinase) released out of the neutrophil after activation. These AMPs were found to be effective not only against bacterial species but also against viruses, fungi and other microorganisms [38,39].

**Figure 1:**
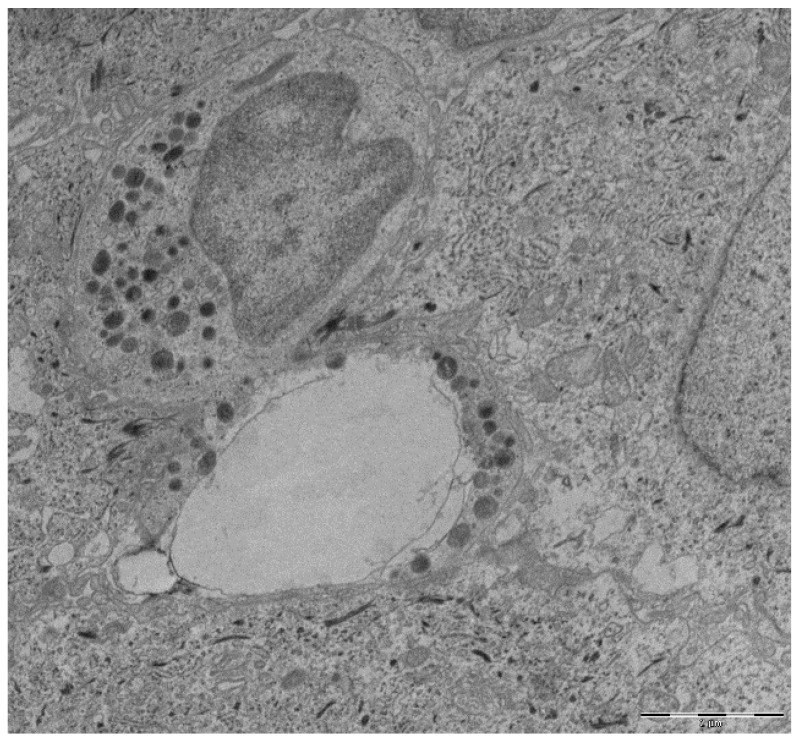
Transmission electron microscopy micrograph from a gingival tissue sample with periodontitis. An emptied disrupted neutrophil alongside an intact one are shown. Scale bar, 2¦Ìm.

Many stimuli have been revealed to induce NET formation, such as viruses, fungi, parasites and host-derived components, such as cytokines and activated platelets [40]. Three main forms of NET formation have been identified. The classical form of NET formation is defined as a programmed cell death, different from necrosis and apoptosis, characterized by disruption of the nuclear membrane that lasts from two to four hours, which gives neutrophils the ability to fight pathogens beyond their lifespan. NET formation starts with the recognition of several stimuli (e.g., bacteria, fungi, viruses) through neutrophil receptors (such as toll-like receptors (TLRs), IgG-Fc receptors and cytokine receptors) [41]. Then, the mobilization of stored calcium ions from the endoplasmic reticulum would also be crucial for the process, the calcium being necessary for the citrullination of the histones and for the activation of protein-arginine deiminase 4 (PAD4) and the release of ROS [42]. The histone deamination by PAD4 is known as a major event in the decondensation of chromatin and the release of NET. ROS play an essential part in promoting the breakdown of the nuclear membrane. NE and deferoxamine are involved in the further decondensation of the nuclear chromatin phenomenon [43]. In addition, NE and myeloperoxidase are dismissed from azurophilic granules and then translocate into the nucleus. Then the nuclear chromatin is extruded into the extracellular space; suicidal NETosis can be recognized microscopically by the presence of disrupted neutrophils in the tissue (Figure 1). NET extrusion from cell death would cause damage of periodontal tissues through an autoimmune phenomenon [44]. However, in 2012, Pilsczek et al. offered another mechanism and stated that the neutrophils formed NETs during highly developed infection with *Staphylococcus aureus* (*S. aureus*), but the neutrophils are still viable, and have the normal function of vital neutrophils in terms of phagocytosis and other purposes. NET formation involves the use of vesicles that carry the chromatin without extracellular release of DNA [45]. This phenomenon is very rapid; it takes place between 5 and 60 min after stimulation and does not involve NADPH oxidase. In this second form, called vital NET formation, neutrophils create NETs but there is no breakdown of the plasma or nuclear membranes [46]. More recently, NET formation from mitochondrial DNA in viable neutrophils has been described [47]; mitochondrial DNA is released instead of nuclear DNA. Mitochondrial NET formation is not related to cell death but is dependent on ROS formation [47]. Mitochondrial NETs are identified in neutrophils within 15 min when stimulated with C5a or lipopolysaccharide (LPS). These findings are not in line with those of Brinkmann et al., who stated that NET formation leads inexorably to the death of the neutrophil [48]; moreover, it is not clear whether the mitochondrial DNA content would be enough for the amount of DNA detected in the traps [40]. The mechanisms that result in the formation of NETs through the release of mitochondrial DNA or through viable cells are still unknown. Interestingly, mitochondrial NETs may be a faster antimicrobial mechanism, which allows cells to remain viable and to prevent the extrusion of phagocytosed bacteria [49], an event that to our knowledge has not yet been studied in relation with periodontitis.

### 3.2. Microbicidal Effects of NETs

Since 2004, many studies have highlighted the ability of NETs to participate in destroying infectious agents, such as bacteria, parasites, fungi and more recently viruses. Bacteria are powerful stimuli that activate the release of NETs [50]. NETs can trap microorganisms and slow their spread from the initial site of infection, probably through the electrostatic interactions between cationic components of NETs and the anionic surface of the pathogen [48]. NETs can also inactivate the virulence factors of pathogenic microorganisms; whose function is to modify and destroy the host cells. This had already been confirmed in the first evidence on NETs, where extracellular NE as a component of NETs actively targeted bacterial virulence factors of *Shigella* spp., such as the adhesin IcsA protein and the invasion plasmid antigen B. The antimicrobial activity of NETs depends on the structure of the NETs, as it provides a high local concentration of proteins with anti-infectious activity in the direct proximity of the trapped pathogen [33]. These proteins’ proteases include enzymes such as antimicrobial peptides and lysozyme. Histones, the most abundant proteins of NETs, also possess a strong ability to kill microorganisms. NETs are involved in the elimination of Gram-positive and Gram-negative bacteria. Among Gram-positive bacteria, *S. aureus* can be destroyed by a mechanism dependent on peroxidase activity of the NET’s MPO [51]. NETs can also kill Gram-negative bacteria, including *Shigella flexneri*, *Escherichia coli* and *Salmonella typhimurium* [34].

### 3.3. Microorganisms’ Strategies to Escape the Action of NETs

Identifying strategies to escape NETs in various microorganisms highlights the long coexistence of neutrophils and infectious agents in evolution, as well as the importance of this mechanism for combating infections [50]. Among these strategies, some bacteria produce DNases and other extracellular nucleases in order to destroy the DNA backbone of NETs and therefore evade this mechanism. This has been demonstrated with *S. aureus* [52] and *Streptococcus pneumonia* (*S. Pneumonia*) [53]. DNase production has been reported by a wide range of periodontal bacterial species and this expression appeared to be a trait in species that have been classically considered as periodontal pathogens, such as species from red (*Porphyromonas gingivalis* (*P. gingivalis*) and *Tannerella forsythia* (*T. forsythia*)), orange (*Fusobacterium nucleatum* (*F. nucleatum*), *Prevotella intermedia* (*P. intermedia*) *and Prevotella nigrescens* (*P. nigrescens*)) and yellow (*Streptococcus gordonii* (*S. gordonii*)) microbial complexes. As *P. gingivalis* is one of the most important periodontal pathogens, the DNase expression of six different strains was analyzed, showing all of them had different degrees of DNase activity [54]. *P. gingivalis* is a potent inducer of NET formation that is mediated by gingipains, but its proteolytical activity has shown to inactivate the bactericidal components of NETs through the activation of protease-activated receptor-2 [55]. Several mutant and wild-type strains of *P. gingivalis* have been analyzed and their results showed that mutant strains induced a characteristic NET formation [56]. *P. intermedia* has also shown a strong nuclease activity when compared with other periodontal bacterial species, suggesting that this species could have a major role in the biofilm ability to evade the action of NETs. In the same study, another major periodontal pathogen such as *Aggregatibacter actinomycetemcomitans* (*A. actinomycetemcomitans*) showed no DNase activity [57].

### 3.4. Removal of NETs

Many investigations about the removal of NETs have been published recently. While the investigations appreciated that the removal of NETs is essential for tissue homeostasis, the processes involved and time required in removing NETs are not well understood. In 2010, it was reported that NETs produced in vitro were stable for over 90 h. DNase 1 is one of the mechanisms responsible for NET degradation, and the presence of DNase 1 inhibitors or anti-NET antibodies that also blocked the access of the enzyme would be responsible for the removal of impaired NETs in cases of autoimmune diseases such as systemic lupus erythematosus [58]. NETs are degraded by macrophages through lysosomic action. However, the whole specific nuclease pathway involved in this process remains difficult to find. A key to this process is that the mechanism of NET removal is similar to that of apoptosis, whereby macrophages do not release pro-inflammatory cytokines [59]. Recently it has been reported that NET degradation is increased in treated periodontitis patients, what indicates that NET degradation contributes to a decreased pro-inflammatory state [60,61].

## 4. NETs and Periodontitis

### 4.1. NETs in Periodontitis Studies

In Table 1, we summarize the articles to date that have studied the role of NETs in periodontitis.

**Figure 2:**
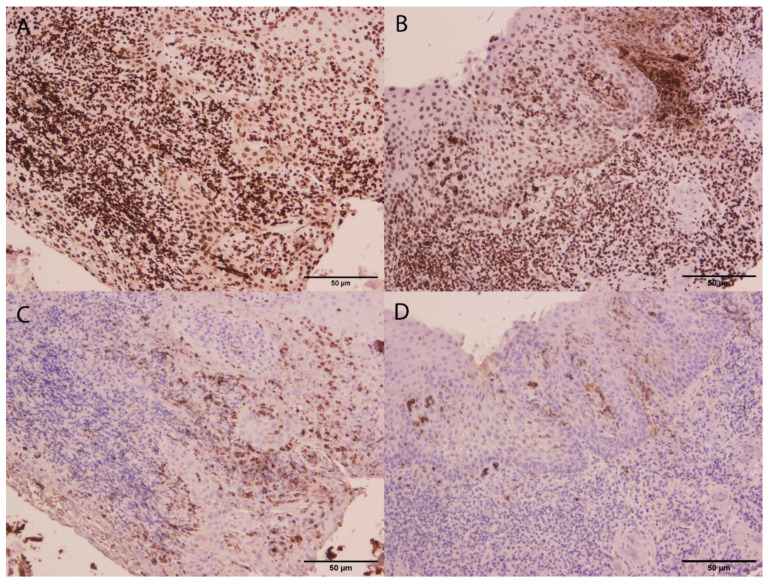
Micrographs from gingivitis (**A**,**C**) and periodontitis (**B**,**D**) gingival tissue samples. Immunostaining of citrullinated histone H3 (**A**,**B**) and MPO (**C**,**D**) are shown. Although citrullinated histone H3 expression did not differ between gingivitis and periodontitis (**A**,**B**), a higher MPO expression in gingivitis compared to periodontitis was found. This suggested that NET formation might be more associated with gingivitis. Scale bar, 50 µm.

With regard to previous results published by our group, we were able to characterize NETs in tissue samples with periodontitis and gingivitis using immunofluorescence, immunohistochemistry and electron microscopy analysis (Figure 2). The comparison of periodontitis and gingivitis showed that NET composition changed, and the general expression of citrullinated histone H3 was found to be higher in gingivitis. These findings suggested that the potential role of NETs in periodontitis may be associated with early and more acute phases of the inflammatory process [63].

Previous investigations (see Table 1) showed that periodontitis led to an increased formation of ROS and NETs. In addition, interferon alpha (IFN-α) was found in significant amounts in periodontitis patients. This mediator is very important for stimulating NET formation and the periodontal pocket provides ideal O_2_ levels and pH for ROS formation [75]. Thus, for all the previous reasons this provides a friendly environment for ROS formation. Investigations suggest that the loss of bone and progression of disease depend on the nature of the inflammatory response of the patient and the type of pathogen.

### 4.2. Microbial Agents Alter NET Formation

Lipopolysaccharide is a key component of Gram-negative bacterial cell walls, where it maintains the structural integrity, stability and negative charge of the bacteria. LPS does not have the capacity to directly induce neutrophils to release NETs; however, there is a growing belief that LPS can activate platelets, which subsequently initiate NET release. It has recently been discovered that TLR4 is present on platelets, which is indicative of platelets having the capacity to recognize and respond to LPS from Gram-negative bacteria [76]. Early studies identified *P. gingivalis*, *Agregatibacter actinomycetemcomitans* and *Tannerella forsythia* as causative agents in periodontal disease and found them to be involved in NET-related processes [77]. NET formation is dependent on the activation of protease-activated receptor 2 (PAR2) by *P. gingivalis*-derived proteases. *P. gingivalis* is found in the oral cavity, where it is implicated in periodontal disease. Furthermore, a novel role has also been demonstrated for proteases as bacterial virulence factors antagonizing the antibacterial activity of NETs [55]. Additionally, the suggested generation of NETs in the periodontium leads to increased inflammation and can be considered another virulence strategy used by *P. gingivalis*. The presentation of intracellular self-antigens modified by gingipains may have immunological consequences, as the excessive presentation of cryptic antigens creates a developed part of systemic diseases associated with periodontitis [78]. Hirschfeld et al. indicated that some bacteria (*Propionibacterium acnes*, *Veillonella parvula* and *Streptococcus gordonii*) led to an enhancement of NET-derived DNA production, via NADPH oxidase-independent mechanisms [71]. It was previously mentioned in this review that NET formation depends strongly on the formation of ROS for its release. Periodontal bacteria produce DNases that reduce NET release levels, and pathogen colonization might increase in the periodontal tissue. Most aggressive pathogens release DNase, disseminating NET contents that lead to the liberation of their antimicrobial components in the surrounding tissue, resulting in a harmful effect on periodontal tissue [54].

As neutrophils are the major and first immune cell to reach the infected area, they are involved in the initial steps of the inflammatory response. Therefore, neutrophils are a determinant component of the immune response in periodontal status [15]. It is reasonable to assume that NET production or effectiveness in periodontitis may be reduced, a reduction in the effectiveness of the NET function would allow easier bacterial infiltration of periodontal tissues, leading to more inflammatory response in the infected area and resulting in tissue destruction. The digestion of NETs via DNase leads to the liberation of NET-associated antimicrobial peptides, which in turn leads to more tissue destruction [44].

### 4.3. Defective Neutrophils and Impaired NET Formation in Periodontitis

Previous evidence has already shown that neutrophils show hyperactivity to bacterial species found in subgingival plaque and an upregulated ROS release [15,79]. Neutrophils in healthy periodontal tissue are moved towards dental biofilms, in which they are stimulated by oral bacteria and their components to form NETs. The migrated oral neutrophil is a viable cell with a hyperactive phenotype, as evidenced by the increased adhesion and internalization of microbes and 13 times more NET formation capacity than the circulating neutrophils [18]. In 2017, Hirschfeld et al. suggested that the variability in neutrophils, such as deficiencies in the number or abnormal function of neutrophils toward various bacteria, might contribute to the pathogenesis of periodontal disease [71]. Periodontitis patients presented with over four times higher oral neutrophil counts compared to healthy periodontal tissue, which was a predictor for protease activity. More oral neutrophils were apoptotic in periodontitis patients than in healthy ones [80,81]. The neutrophil-mediated antimicrobial action fails to stop the bacteria in cases of periodontitis, leading to tissue damage and destruction of both bacterial and immune origin. NET formation is also considered a potential factor changing the influence of the individual course of periodontitis [82]. Periodontitis in Papillon–Lefèvre (PLS) syndrome arises from the failure to eliminate periodontal pathogens because of cathepsin C deficiency [83]. PLS neutrophils reduced the capacity for NET production, characterized by the absence of the NET-related proteins such as chorionic gonadotropin, MPO and NE. ROS formation was higher in PLS [72]. The failure of activities of neutrophil antimicrobial proteins might maintain the stimulus for the wrongful recruiting of highly responsive neutrophils in periodontal tissues, providing a reasonable explanation for the acute inflammation and bone loss that characterize PLS periodontitis patients [84,85]. Interestingly, individuals with PLS do not suffer any systemic infections—rarely are there any skin abscesses. Therefore, the defects of neutrophils appear to be localized in areas of the human body more susceptible to a direct and chronic bacterial challenge, such as the oral cavity [86].

This hyper-reactivity may come from the excessive NET formation in response to periodontal pathogens and/or local mediators [66]. The implication of the neutrophils and their enzymes is supported by the fact that high levels of NETs remain in the tissue for an extended period. In addition, this supports the hypothesis that NET formation is dependent on ROS formation, which has been shown to be higher in periodontitis [15]. The neutrophil function in periodontitis may be a key determinant of the patient’s periodontal health status.

**Figure 3:**
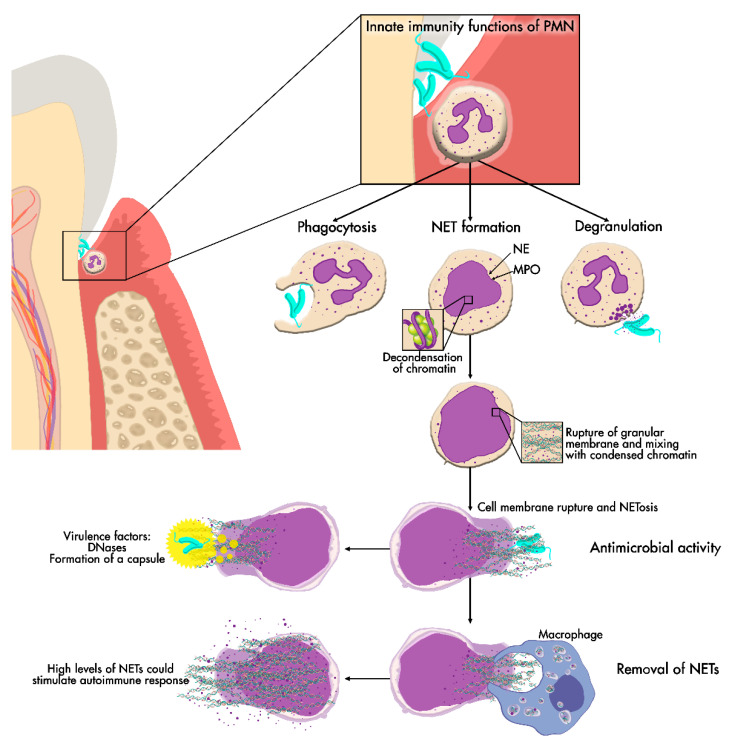
NET release. NET formation may be one of the main neutrophil functions in periodontal tissue. NET production starts with chromatin decondensation, which is then embedded with cytoplasmic antimicrobial peptide granules. NETs are then released into the extracellular space after cell membrane rupture to exert their antimicrobial effect and later removed from the tissue. If NET removal fails, persistent high levels of NETs could cause damage to periodontal tissues.

In addition, increased neutrophil ROS formation is associated with elevated IFNα levels in periodontitis, indicating that this class of signaling proteins is also important in NET formation [75,87]. High levels of NETs within periodontal tissue could stimulate an autoimmune response, resulting in augmented neutrophil levels and causing more tissue destruction [73]. This hypothesis of NETs’ hyperactivity in periodontitis is supported by Vitkov et al. They investigated NETs in exudate samples from the gingiva of periodontitis patients and compared the results with previous examinations of abscesses. In addition, they found that the samples collected had high levels of NETs and that in seven samples 22 trapped bacteria were associated with the NETs. In addition, based on the use of electron microscopy and analysis of gingival biopsies, patients with chronic periodontitis showed the presence of NETs [69]. In a recent study from the same authors, they hypothesize that there is a dissemination phenomenon of bacterial species, LPS and antigens citrullinated by NETs from the infected periodontal tissue. This dissemination could contribute to exacerbated autoimmune diseases such as RA via the activation of TLR receptors [78]. Therefore, both mechanisms of NET formation may be responsible for tissue destruction [88]. The impaired degradation of NETs and the escape of pathogens from the effect of NETs by virulence factors leads to a response from neutrophils, upregulating the release of NETs, resulting in the immobilization and localization of neutrophils instead of trapping bacteria, which leads to tissue destruction [34] (Figure 3).

## 5. Role of NETs in Systemic Diseases

The formation of NETs could promote thrombosis via histones [61]. NETs in combination with platelets may damage the blood vessels during sepsis, destroying endothelial cells and causing vascular occlusions [76,89]. On the other hand, it has been reported that NETs might promote the implantation of metastases through the uptake of circulating malignant cells [90]. Garley et al. indicated in 2018 that the neutrophils of patients with oral inflammation with stage I/II cancer produce increased formation of NETs compared to the neutrophils of healthy humans. However, the amount of NETs in stage III/IV cancer patients was lower than the amount of NETs in inflammation and early-stage cancer patients [91].

NETs have been described as a source of auto-antigens in various autoimmune diseases, such as vasculitis, lupus, psoriasis and RA [92]. NETs exhibit proteins normally restricted to the interior of the granules, nucleus or cytoplasm. This exposure would result in immunization against self-antigens and create autoimmune disorders. For example, in anti-neutrophil cytoplasmic antibody-associated vasculitis, proteinase 3 and MPO are self-antigens targeted by auto-antibodies, and these two enzymes are associated with NETs [93]. NETs have also been shown to have adverse effects in pre-eclampsia, where placenta-derived cytokine-activated neutrophils activated NET extrusion. NETs were found in the intervillous space of placental tissue samples [94]. In atherosclerosis, dendritic cell activation by NETs is similar to that which occurs in lupus, and these dendritic cells are one of the cell populations found in atheromatous plaques [95].

### The Relationship between Rheumatoid Arthritis, Periodontitis and NETs

Periodontitis and RA are considered to be two chronic inflammatory diseases with a common pathogenesis. RA is an autoimmune inflammatory disease defined by the destruction and inflammation of joints and internal organs in which citrullination is a central feature leading to the generation of auto-antibodies to citrullinated protein antigens. In periodontitis, citrullination either by NET formation or *P. gingivalis*-derived peptydil arginine deiminase activity has been suggested [96], although citrullination independent of oral bacteria has also been reported [97]. Evidence has suggested that citrullinated antigens in RA are mostly derived from NETs [98]. Patients with periodontitis may have RA and vice versa [99]. A recent publication has suggested that periodontal indices such as gingival index, probing pocket depth (PPD) and bleeding on probing (BOP) have positive relationships with RA. Anti-*P. gingivalis* antibody levels were associated with BOP, PPD and GI and the severity of periodontitis; thus, increasing the values of periodontitis indices could be a sign of advanced disease development in RA patients [100]. In addition, a high level of anti-*P. gingivalis* antibody could be regarded as a warning sign in RA patients suffering from periodontitis [101]. Non-surgical periodontal treatment has shown to improve symptoms in both diseases [102,103,104]. Previous studies demonstrated that NETs were increased in the synovial fluid, rheumatoid nodules, peripheral blood and skin of RA patients [92]. Increased NET formation in the oral cavity of periodontitis patients perhaps plays a part in the initiation of RA [105]. *P. gingivalis* is the most important pathogen responsible for periodontitis. Further, it was shown that *P. gingivalis* could induce NET generation [55]. Interestingly, a study has demonstrated that patients with periodontitis and RA showed significantly higher serum levels of NETs than the control group. Furthermore, a periodontal cure remarkably decreased the serum levels of NETs in patients with RA and periodontitis [65]. However, more studies are required with a greater number of cases and a longer evolution time in order to understand the relation between the two diseases.

## 6. Conclusions and Future Research Lines

NETs trap and/or kill a wide variety of microorganisms, bacteria, fungi and parasites through their antimicrobial agents, such as MPO, NE and proteinase. NET formation has been associated with different diseases, such as inflammatory diseases including periodontitis and autoimmune diseases such as RA. Excess formation of NETs can be harmful to periodontal tissue if they are not correctly removed; consequently, increased NET degradation has been reported following periodontal treatment. As stated by a recently published consensus document, several areas regarding the study of NETs are still controversial. Specifically, the origin of the DNA found in NETs should be identified in order to find a clear way to distinguish NET formation from other forms of programmed cell death, and to identify all the pathways that regulate NET formation, since it is very unlikely that it is mediated by a single pathway. There is also a great need for standardization of the methodologies used for the identification of NETs [37]. Finally, NETs are currently considered potential therapeutic targets. Treatment with Nupharidine, an agent purified from the plant *Nuphar lutea*, has been shown to increase NET extrusions by neutrophil-like cells by 106%. However, the authors claim that whether the increase in NET extrusion by this compound has a detrimental or protective effect on the periodontal tissues requires further in vivo research [64]. Therefore, NETs can be considered as potential therapeutic targets for periodontitis as well as for other diseases of autoimmune origin. Certainly, the role of NETs in periodontitis needs to be further studied to enable a full understanding of their role in the pathogenicity of the disease.

## Figures and Tables

**Table 1:** Summary table of the studies assessing the role of neutrophil extracellular traps (NETs) in periodontitis and the induction of NET formation by periodontal bacteria.

**Studies on the Expression of NETs in Periodontitis Patients**					
Author	Year	Participants	Types of samples	NET marker	Results
Zhang et al. [62]	2020	27 periodontitis, 17 gingivitis and 20 controls	Peripheral blood neutrophils	IL-8 and TNF-alpha as NETs inducers	Periodontitis showed lower expression of IL-8 compared to controls
Moonen et al. A [61]	2019	1st part:38 periodontitis and 38 controls 2nd part: 91 periodontitis before and after treatment	Peripheral blood neutrophils	SYTOX Green	No differences in NET degradation between healthy subjects and periodontitis. Periodontal therapy increased NET degradation
Magán-Fernández et al. [63]	2019	6 Chronic periodontitis, 5 gingivitis and 2 controls	Gingival tissue biopsies	CitH3 and MPO	Higher H3 in gingivitis and MPO higher in periodontitis
Levy et al. [64]	2019	3 Localized aggressive periodontitis and 3 controls and HL60 neutrophils	Peripheral blood neutrophils and HL60 neutrophils incubated with nupharidine	SYTOX Green	NET formation was higher in the neutrophils exposed to Nupharidine
Kaneko et al. [65]	2018	40 Rheumatoid arthritis and periodontitis, 30 periodontitis and 43 controls	Serum samples	NET-associated MPO-DNA complexes by ELISA	NETs increased in the RA + periodontitis group. NETs were associated with moderate to severe periodontitis. Periodontal treatment reduced NETs
White et al. [60]	2016	Chronic periodontitis and controls (40 pairs)	Peripheral blood neutrophils stimulated with PMA or HOCl	SYTOX Green	NET formation decreased and NET removal was restored following periodontal treatment
Fine et al. [66]	2016	17 Chronic periodontitis and 11 controls	Blood and oral neutrophils	CitH3, MPO, CD18	Proinflammatory oral neutrophils from periodontitis showed high levels of NET formation compared to controls
Hirschfeld et al. [67]	2015	14 Experimental gingivitis and 6 controls	Supragingival plaque, peripheral blood neutrophils	CitH3, Histone H1, CD-177, MPO, NE, Cathepsin-G.	NETs were found within the oral biofilm. Bacterial isolates tested induced NET formation.
Vitkov et al. [68]	2010	26 Periodontitis	GCF (18); Purulent crevicular exudate (8)	Scanning electron microscopy (SEM); CitH3 and DNA	All neutrophils in the samples were citrullinated. 78% of them showed dispersed NETs
Vitkov et al. [69]	2009	22 Chronic Periodontitis	Purulent crevicular exudate (22); Gingival biopsies (12)	Exudates: NE and DNA; Biopsies: Transmission electron microscopy (TEM) and SEM (with and without DNase).	NETs were found on all the exudate samples. DNase caused the disappearance of NETs
**In Vitro Studies on NET Formation Induced by Periodontal Bacteria**					
Author	Year	Participants	Types of samples	NET marker	Results
Bryzek et al. [55]	2019	Human donors	Peripheral blood neutrophils stimulated with different *P. gingivalis* strains, antigens and gingipains	NE, Hoechst 33342, ADNbc PicoGreen^®^ and DNase I	Gingipains from *P. gingivalis* induce NETs formation and prevent *P. gingivalis* entrapment and killing
Alyami et al. [70]	2019	In vitro PMN layers	Human primary neutrophils infected with *Aggregatibacter actinomycetemcomitans, P. gingivalis and F. nucleatum*	SYTOX Orange, NE, CitH3, DAPI	*F.nucleatum* induced rapid and robust NET formation trough NOD1 and NOD 2 receptors
Hirschfeld et al. [71]	2017	10 Healthy donors	Peripheral blood neutrophils. Stimulation with 19 periodontal bacteria	FITC NET-DNA, NE, and MPO	Certain species stimulated higher NET formation.
Doke et al. [57]	2017	Healthy donors	PMA-stimulated peripheral blood neutrophils. Nucleases from several periodontal bacteria.	SYTOX Orange, NE and DAPI	*Prevotella intermedia* demonstrated the highest NET degradation of all the Gram—periodontal bacteria
Roberts et al. [72]	2016	5 Papillon–Lefévre syndrome (PLS) patients and 5 controls	Peripheral blood neutrophils stimulated with periodontal bacteria	SYTOX Green, NE, NET-bound MPO, NET-bound CG	Neutrophils from PLS patients have a reduced capacity for NET formation and a compromised antimicrobial activity
Palmer et al. [73]	2016	Healthy donors	Peripheral blood neutrophils incubated with oral bacteria in different complement blocking conditions	NET-DNA fluorometry	Complement and IgG enhance NET formation by several periodontal bacteria
Hirschfeld et al. [74]	2016	Healthy donors	Peripheral blood neutrophils with A.a., A.a. leucotoxin	Micrococcal nuclease	The leucotoxic strain of A.a. and high concentrations of A.a. leucotoxin induced NET formation
Jayaprakash et al. [56]	2015	Healthy donors	In vitro PMA-generated NETs;	FITC-labeled *P. gingivalis*, F-actin, DNA	*P. gingivalis* strains K1A and E8 induced NET formation
Palmer et al. [54]	2012	Healthy donors	In vitro PMA-generated NETs	DNase activity of periodontal bacterial species. SYTOX Green	DNase producing species caused the degradation of NETs
**Other Studies Regarding NET Formation in Oral Neutrophils**					
Moonen et al. [18]	2019	9 Healthy donors	PMA-stimulated venous blood neutrophils and oral neutrophils	SYTOX Green	Oral neutrophils showed greater NET formation than circulating neutrophils in both stimulated and non-stimulated groups

## References

[1] Lamont R.J., Koo H., Hajishengallis G. (2018). The oral microbiota: Dynamic communities and host interactions. Nat. Rev. Microbiol..

[2] Darveau R.P. (2010). Periodontitis: A polymicrobial disruption of host homeostasis. Nat. Rev. Microbiol..

[3] Nibali L., Henderson B., Sadiq S.T., Donos N. (2014). Genetic dysbiosis: The role of microbial insults in chronic inflammatory diseases. J. Oral. Microbiol..

[4] Nauseef W.M. (2014). Proteases, neutrophils, and periodontitis: The net effect. J. Clin. Investig..

[5] Bascones-Martinez A., Munoz-Corcuera M., Noronha S., Mota P., Bascones-Ilundain C., Campo-Trapero J. (2009). Host defence mechanisms against bacterial aggression in periodontal disease: Basic mechanisms. Med. Oral Patol. Oral Cir. Bucal..

[6] Kassebaum N.J., Bernabe E., Dahiya M., Bhandari B., Murray C.J., Marcenes W. (2014). Global burden of severe periodontitis in 1990–2010: A systematic review and meta-regression. J. Dent. Res..

[7] Frencken J.E., Sharma P., Stenhouse L., Green D., Laverty D., Dietrich T. (2017). Global epidemiology of dental caries and severe periodontitis—A comprehensive review. J. Clin. Periodontol..

[8] Marcenes W., Kassebaum N.J., Bernabe E., Flaxman A., Naghavi M., Lopez A., Murray C.J. (2013). Global burden of oral conditions in 1990–2010: A systematic analysis. J. Dent. Res..

[9] Eke P.I., Dye B.A., Wei L., Slade G.D., Thornton-Evans G.O., Borgnakke W.S., Taylor G.W., Page R.C., Beck J.D., Genco R.J. (2015). Update on prevalence of periodontitis in adults in the united states: Nhanes 2009 to 2012. J. Periodontol..

[10] Mesa F., Liebana J., Galindo-Moreno P., J. O’Valle F. (2011). Oral pathogens, immunity, and periodontal diseases. Curr. Immunol. Rev..

[11] Jaillon S., Galdiero M.R., Del Prete D., Cassatella M.A., Garlanda C., Mantovani A. (2013). Neutrophils in innate and adaptive immunity. Semin. Immunopathol..

[12] Scott D.A., Krauss J. (2012). Neutrophils in periodontal inflammation. Front. Oral Biol..

[13] Cortes-Vieyra R., Rosales C., Uribe-Querol E. (2016). Neutrophil functions in periodontal homeostasis. J. Immunol. Res..

[14] De Smit M., Westra J., Vissink A., Doornbos-van der Meer B., Brouwer E., van Winkelhoff A.J. (2012). Periodontitis in established rheumatoid arthritis patients: A cross-sectional clinical, microbiological and serological study. Arthritis Res. Ther..

[15] Matthews J.B., Wright H.J., Roberts A., Ling-Mountford N., Cooper P.R., Chapple I.L. (2007). Neutrophil hyper-responsiveness in periodontitis. J. Dent. Res..

[16] Eash K.J., Greenbaum A.M., Gopalan P.K., Link D.C. (2010). Cxcr2 and cxcr4 antagonistically regulate neutrophil trafficking from murine bone marrow. J. Clin. Investig..

[17] Thorbert-Mros S., Larsson L., Berglundh T. (2015). Cellular composition of long-standing gingivitis and periodontitis lesions. J. Periodontal. Res..

[18] Moonen C.G.J., Hirschfeld J., Cheng L., Chapple I.L.C., Loos B.G., Nicu E.A. (2019). Oral neutrophils characterized: Chemotactic, phagocytic, and neutrophil extracellular trap (net) formation properties. Front. Immunol..

[19] Hirschfeld J. (2019). Neutrophil subsets in periodontal health and disease: A mini review. Front. Immunol..

[20] Zeidler C., Germeshausen M., Klein C., Welte K. (2009). Clinical implications of ela2-, hax1-, and g-csf-receptor (csf3r) mutations in severe congenital neutropenia. Br. J. Haematol..

[21] Mc Guire P.J., Cunningham-Rundles C., Ochs H., Diaz G.A. (2010). Oligoclonality, impaired class switch and b-cell memory responses in whim syndrome. Clin. Immunol..

[22] Hajishengallis E., Hajishengallis G. (2014). Neutrophil homeostasis and periodontal health in children and adults. J. Dent. Res..

[23] Silva L.M., Brenchley L., Moutsopoulos N.M. (2019). Primary immunodeficiencies reveal the essential role of tissue neutrophils in periodontitis. Immunol. Rev..

[24] Hajishengallis G. (2020). New developments in neutrophil biology and periodontitis. Periodontology 2000.

[25] Almarza Novoa E., Kasbekar S., Thrasher A.J., Kohn D.B., Sevilla J., Nguyen T., Schwartz J.D., Bueren J.A. (2018). Leukocyte adhesion deficiency-i: A comprehensive review of all published cases. J. Allergy Clin. Immunol. Pract..

[26] Hanna S., Etzioni A. (2012). Leukocyte adhesion deficiencies. Ann. N. Y. Acad. Sci..

[27] Choi E.Y., Chavakis E., Czabanka M.A., Langer H.F., Fraemohs L., Economopoulou M., Kundu R.K., Orlandi A., Zheng Y.Y., Prieto D.A. (2008). Del-1, an endogenous leukocyte-endothelial adhesion inhibitor, limits inflammatory cell recruitment. Science.

[28] Kourtzelis I., Li X., Mitroulis I., Grosser D., Kajikawa T., Wang B., Grzybek M., von Renesse J., Czogalla A., Troullinaki M. (2019). Del-1 promotes macrophage efferocytosis and clearance of inflammation. Nat. Immunol..

[29] Roberts H.M., Ling M.R., Insall R., Kalna G., Spengler J., Grant M.M., Chapple I.L. (2015). Impaired neutrophil directional chemotactic accuracy in chronic periodontitis patients. J. Clin. Periodontol..

[30] Moutsopoulos N.M., Konkel J., Sarmadi M., Eskan M.A., Wild T., Dutzan N., Abusleme L., Zenobia C., Hosur K.B., Abe T. (2014). Defective neutrophil recruitment in leukocyte adhesion deficiency type i disease causes local il-17-driven inflammatory bone loss. Sci. Transl. Med..

[31] Moutsopoulos N.M., Zerbe C.S., Wild T., Dutzan N., Brenchley L., DiPasquale G., Uzel G., Axelrod K.C., Lisco A., Notarangelo L.D. (2017). Interleukin-12 and interleukin-23 blockade in leukocyte adhesion deficiency type 1. N. Engl. J. Med..

[32] Nakazawa D., Kumar S., Desai J., Anders H.J. (2017). Neutrophil extracellular traps in tissue pathology. Histol. Histopathol..

[33] Brinkmann V., Reichard U., Goosmann C., Fauler B., Uhlemann Y., Weiss D.S., Weinrauch Y., Zychlinsky A. (2004). Neutrophil extracellular traps kill bacteria. Science.

[34] Delgado-Rizo V., Martinez-Guzman M.A., Iniguez-Gutierrez L., Garcia-Orozco A., Alvarado-Navarro A., Fafutis-Morris M. (2017). Neutrophil extracellular traps and its implications in inflammation: An overview. Front. Immunol..

[35] Desai J., Kumar S.V., Mulay S.R., Konrad L., Romoli S., Schauer C., Herrmann M., Bilyy R., Muller S., Popper B. (2016). Pma and crystal-induced neutrophil extracellular trap formation involves ripk1-ripk3-mlkl signaling. Eur. J. Immunol..

[36] Galluzzi L., Vitale I., Aaronson S.A., Abrams J.M., Adam D., Agostinis P., Alnemri E.S., Altucci L., Amelio I., Andrews D.W. (2018). Molecular mechanisms of cell death: Recommendations of the nomenclature committee on cell death 2018. Cell Death Differ..

[37] Boeltz S., Amini P., Anders H.J., Andrade F., Bilyy R., Chatfield S., Cichon I., Clancy D.M., Desai J., Dumych T. (2019). To net or not to net:Current opinions and state of the science regarding the formation of neutrophil extracellular traps. Cell Death Differ..

[38] Sierra J.M., Fuste E., Rabanal F., Vinuesa T., Vinas M. (2017). An overview of antimicrobial peptides and the latest advances in their development. Expert Opin. Biol. Ther..

[39] Kang H.K., Kim C., Seo C.H., Park Y. (2017). The therapeutic applications of antimicrobial peptides (amps): A patent review. J. Microbiol..

[40] Brinkmann V. (2018). Neutrophil extracellular traps in the second decade. J. Innate Immun..

[41] Fuchs T.A., Abed U., Goosmann C., Hurwitz R., Schulze I., Wahn V., Weinrauch Y., Brinkmann V., Zychlinsky A. (2007). Novel cell death program leads to neutrophil extracellular traps. J. Cell Biol..

[42] Gupta A.K., Giaglis S., Hasler P., Hahn S. (2014). Efficient neutrophil extracellular trap induction requires mobilization of both intracellular and extracellular calcium pools and is modulated by cyclosporine a. PLoS ONE.

[43] Papayannopoulos V., Metzler K.D., Hakkim A., Zychlinsky A. (2010). Neutrophil elastase and myeloperoxidase regulate the formation of neutrophil extracellular traps. J. Cell Biol..

[44] Cooper P.R., Palmer L.J., Chapple I.L. (2013). Neutrophil extracellular traps as a new paradigm in innate immunity: Friend or foe?. Periodontology 2000.

[45] Pilsczek F.H., Salina D., Poon K.K., Fahey C., Yipp B.G., Sibley C.D., Robbins S.M., Green F.H., Surette M.G., Sugai M. (2010). A novel mechanism of rapid nuclear neutrophil extracellular trap formation in response to staphylococcus aureus. J. Immunol..

[46] Li P., Li M., Lindberg M.R., Kennett M.J., Xiong N., Wang Y. (2010). Pad4 is essential for antibacterial innate immunity mediated by neutrophil extracellular traps. J. Exp. Med..

[47] Yousefi S., Mihalache C., Kozlowski E., Schmid I., Simon H.U. (2009). Viable neutrophils release mitochondrial DNA to form neutrophil extracellular traps. Cell Death Differ..

[48] Brinkmann V., Zychlinsky A. (2007). Beneficial suicide: Why neutrophils die to make nets. Nat. Rev. Microbiol..

[49] Jorch S.K., Kubes P. (2017). An emerging role for neutrophil extracellular traps in noninfectious disease. Nat. Med..

[50] Arazna M., Pruchniak M.P., Demkow U. (2013). Neutrophil extracellular traps in bacterial infections: Strategies for escaping from killing. Respir. Physiol. Neurobiol..

[51] Parker H., Albrett A.M., Kettle A.J., Winterbourn C.C. (2012). Myeloperoxidase associated with neutrophil extracellular traps is active and mediates bacterial killing in the presence of hydrogen peroxide. J. Leukoc. Biol..

[52] Berends E.T., Horswill A.R., Haste N.M., Monestier M., Nizet V., von Kockritz-Blickwede M. (2010). Nuclease expression by staphylococcus aureus facilitates escape from neutrophil extracellular traps. J. Innate Immun..

[53] Beiter K., Wartha F., Albiger B., Normark S., Zychlinsky A., Henriques-Normark B. (2006). An endonuclease allows streptococcus pneumoniae to escape from neutrophil extracellular traps. Curr. Biol..

[54] Palmer L.J., Chapple I.L., Wright H.J., Roberts A., Cooper P.R. (2012). Extracellular deoxyribonuclease production by periodontal bacteria. J. Periodontal. Res..

[55] Bryzek D., Ciaston I., Dobosz E., Gasiorek A., Makarska A., Sarna M., Eick S., Puklo M., Lech M., Potempa B. (2019). Triggering netosis via protease-activated receptor (par)-2 signaling as a mechanism of hijacking neutrophils function for pathogen benefits. PLoS Pathog..

[56] Jayaprakash K., Demirel I., Khalaf H., Bengtsson T. (2015). The role of phagocytosis, oxidative burst and neutrophil extracellular traps in the interaction between neutrophils and the periodontal pathogen porphyromonas gingivalis. Mol. Oral Microbiol..

[57] Doke M., Fukamachi H., Morisaki H., Arimoto T., Kataoka H., Kuwata H. (2017). Nucleases from prevotella intermedia can degrade neutrophil extracellular traps. Mol. Oral Microbiol..

[58] Hakkim A., Furnrohr B.G., Amann K., Laube B., Abed U.A., Brinkmann V., Herrmann M., Voll R.E., Zychlinsky A. (2010). Impairment of neutrophil extracellular trap degradation is associated with lupus nephritis. Proc. Natl. Acad. Sci. USA.

[59] Farrera C., Fadeel B. (2013). Macrophage clearance of neutrophil extracellular traps is a silent process. J. Immunol..

[60] White P., Sakellari D., Roberts H., Risafi I., Ling M., Cooper P., Milward M., Chapple I. (2016). Peripheral blood neutrophil extracellular trap production and degradation in chronic periodontitis. J. Clin. Periodontol..

[61] Moonen C.G., Buurma K.G., Faruque M.R., Balta M.G., Liefferink E., Bizzarro S., Nicu E.A., Loos B.G. (2019). Periodontal therapy increases neutrophil extracellular trap degradation. Innate Immun..

[62] Zhang F., Yang X.M., Jia S.Y. (2020). Characteristics of neutrophil extracellular traps in patients with periodontitis and gingivitis. Braz. Oral Res..

[63] Magan-Fernandez A., O’Valle F., Abadia-Molina F., Munoz R., Puga-Guil P., Mesa F. (2019). Characterization and comparison of neutrophil extracellular traps in gingival samples of periodontitis and gingivitis: A pilot study. J. Periodontal Res..

[64] Levy D.H., Chapple I.L.C., Shapira L., Golan-Goldhirsh A., Gopas J., Polak D. (2019). Nupharidine enhances aggregatibacter actinomycetemcomitans clearance by priming neutrophils and augmenting their effector functions. J. Clin. Periodontol..

[65] Kaneko C., Kobayashi T., Ito S., Sugita N., Murasawa A., Nakazono K., Yoshie H. (2018). Circulating levels of carbamylated protein and neutrophil extracellular traps are associated with periodontitis severity in patients with rheumatoid arthritis: A pilot case-control study. PLoS ONE.

[66] Fine N., Hassanpour S., Borenstein A., Sima C., Oveisi M., Scholey J., Cherney D., Glogauer M. (2016). Distinct oral neutrophil subsets define health and periodontal disease states. J. Dent. Res..

[67] Hirschfeld J., Dommisch H., Skora P., Horvath G., Latz E., Hoerauf A., Waller T., Kawai T., Jepsen S., Deschner J. (2015). Neutrophil extracellular trap formation in supragingival biofilms. Int. J. Med. Microbiol..

[68] Vitkov L., Klappacher M., Hannig M., Krautgartner W.D. (2010). Neutrophil fate in gingival crevicular fluid. Ultrastruct. Pathol..

[69] Vitkov L., Klappacher M., Hannig M., Krautgartner W.D. (2009). Extracellular neutrophil traps in periodontitis. J. Periodontal. Res..

[70] Alyami H.M., Finoti L.S., Teixeira H.S., Aljefri A., Kinane D.F., Benakanakere M.R. (2019). Role of nod1/nod2 receptors in fusobacterium nucleatum mediated netosis. Microb. Pathog..

[71] Hirschfeld J., White P.C., Milward M.R., Cooper P.R., Chapple I.L.C. (2017). Modulation of neutrophil extracellular trap and reactive oxygen species release by periodontal bacteria. Infect. Immun..

[72] Roberts H., White P., Dias I., McKaig S., Veeramachaneni R., Thakker N., Grant M., Chapple I. (2016). Characterization of neutrophil function in papillon-lefevre syndrome. J. Leukoc. Biol..

[73] Palmer L.J., Damgaard C., Holmstrup P., Nielsen C.H. (2016). Influence of complement on neutrophil extracellular trap release induced by bacteria. J. Periodontal Res..

[74] Hirschfeld J., Roberts H.M., Chapple I.L., Parcina M., Jepsen S., Johansson A., Claesson R. (2016). Effects of aggregatibacter actinomycetemcomitans leukotoxin on neutrophil migration and extracellular trap formation. J. Oral Microbiol..

[75] Wright H.J., Matthews J.B., Chapple I.L., Ling-Mountford N., Cooper P.R. (2008). Periodontitis associates with a type 1 ifn signature in peripheral blood neutrophils. J. Immunol..

[76] Clark S.R., Ma A.C., Tavener S.A., McDonald B., Goodarzi Z., Kelly M.M., Patel K.D., Chakrabarti S., McAvoy E., Sinclair G.D. (2007). Platelet tlr4 activates neutrophil extracellular traps to ensnare bacteria in septic blood. Nat. Med..

[77] White P., Cooper P., Milward M., Chapple I. (2014). Differential activation of neutrophil extracellular traps by specific periodontal bacteria. Free Radic. Biol. Med..

[78] Vitkov L., Hannig M., Minnich B., Herrmann M. (2018). Periodontal sources of citrullinated antigens and tlr agonists related to ra. Autoimmunity.

[79] Matthews J.B., Wright H.J., Roberts A., Cooper P.R., Chapple I.L. (2007). Hyperactivity and reactivity of peripheral blood neutrophils in chronic periodontitis. Clin. Exp. Immunol..

[80] Nicu E.A., Rijkschroeff P., Wartewig E., Nazmi K., Loos B.G. (2018). Characterization of oral polymorphonuclear neutrophils in periodontitis patients: A case-control study. BMC Oral Health.

[81] Borenstein A., Fine N., Hassanpour S., Sun C., Oveisi M., Tenenbaum H.C., Glogauer M. (2018). Morphological characterization of para- and proinflammatory neutrophil phenotypes using transmission electron microscopy. J. Periodontal Res..

[82] Cekici A., Kantarci A., Hasturk H., Van Dyke T.E. (2014). Inflammatory and immune pathways in the pathogenesis of periodontal disease. Periodontology 2000.

[83] Bullon P., Castejon-Vega B., Roman-Malo L., Jimenez-Guerrero M.P., Cotan D., Forbes-Hernandez T.Y., Varela-Lopez A., Perez-Pulido A.J., Giampieri F., Quiles J.L. (2018). Autophagic dysfunction in patients with papillon-lefevre syndrome is restored by recombinant cathepsin c treatment. J. Allergy Clin. Immunol..

[84] Jepsen S., Caton J.G., Albandar J.M., Bissada N.F., Bouchard P., Cortellini P., Demirel K., de Sanctis M., Ercoli C., Fan J. (2018). Periodontal manifestations of systemic diseases and developmental and acquired conditions: Consensus report of workgroup 3 of the 2017 world workshop on the classification of periodontal and peri-implant diseases and conditions. J. Periodontol..

[85] Hahn J., Schauer C., Czegley C., Kling L., Petru L., Schmid B., Weidner D., Reinwald C., Biermann M.H.C., Blunder S. (2019). Aggregated neutrophil extracellular traps resolve inflammation by proteolysis of cytokines and chemokines and protection from antiproteases. FASEB J..

[86] Byrd A.S., O’Brien X.M., Johnson C.M., Lavigne L.M., Reichner J.S. (2013). An extracellular matrix-based mechanism of rapid neutrophil extracellular trap formation in response to candida albicans. J. Immunol..

[87] Martinelli S., Urosevic M., Daryadel A., Oberholzer P.A., Baumann C., Fey M.F., Dummer R., Simon H.U., Yousefi S. (2004). Induction of genes mediating interferon-dependent extracellular trap formation during neutrophil differentiation. J. Biol. Chem..

[88] Kaplan M.J., Radic M. (2012). Neutrophil extracellular traps: Double-edged swords of innate immunity. J. Immunol..

[89] McDonald B., Davis R.P., Kim S.J., Tse M., Esmon C.T., Kolaczkowska E., Jenne C.N. (2017). Platelets and neutrophil extracellular traps collaborate to promote intravascular coagulation during sepsis in mice. Blood.

[90] Cools-Lartigue J., Spicer J., McDonald B., Gowing S., Chow S., Giannias B., Bourdeau F., Kubes P., Ferri L. (2013). Neutrophil extracellular traps sequester circulating tumor cells and promote metastasis. J. Clin. Investig..

[91] Garley M., Dziemianczyk-Pakiela D., Grubczak K., Surazynski A., Dabrowska D., Ratajczak-Wrona W., Sawicka-Powierza J., Borys J., Moniuszko M., Palka J.A. (2018). Differences and similarities in the phenomenon of nets formation in oral inflammation and in oral squamous cell carcinoma. J. Cancer.

[92] Khandpur R., Carmona-Rivera C., Vivekanandan-Giri A., Gizinski A., Yalavarthi S., Knight J.S., Friday S., Li S., Patel R.M., Subramanian V. (2013). Nets are a source of citrullinated autoantigens and stimulate inflammatory responses in rheumatoid arthritis. Sci. Transl. Med..

[93] Panda R., Krieger T., Hopf L., Renne T., Haag F., Rober N., Conrad K., Csernok E., Fuchs T.A. (2017). Neutrophil extracellular traps contain selected antigens of anti-neutrophil cytoplasmic antibodies. Front. Immunol..

[94] Gupta A., Hasler P., Gebhardt S., Holzgreve W., Hahn S. (2006). Occurrence of neutrophil extracellular DNA traps (nets) in pre-eclampsia: A link with elevated levels of cell-free DNA?. Ann. NY Acad. Sci..

[95] Drechsler M., Doring Y., Megens R.T., Soehnlein O. (2011). Neutrophilic granulocytes—Promiscuous accelerators of atherosclerosis. Thromb. Haemost..

[96] Olsen I., Singhrao S.K., Potempa J. (2018). Citrullination as a plausible link to periodontitis, rheumatoid arthritis, atherosclerosis and alzheimer’s disease. J. Oral Microbiol..

[97] Engstrom M., Eriksson K., Lee L., Hermansson M., Johansson A., Nicholas A.P., Gerasimcik N., Lundberg K., Klareskog L., Catrina A.I. (2018). Increased citrullination and expression of peptidylarginine deiminases independently of p. Gingivalis and a. Actinomycetemcomitans in gingival tissue of patients with periodontitis. J. Trans. Med..

[98] Corsiero E., Pratesi F., Prediletto E., Bombardieri M., Migliorini P. (2016). Netosis as source of autoantigens in rheumatoid arthritis. Front. Immunol..

[99] Zhao X., Liu Z., Shu D., Xiong Y., He M., Xu S., Si S., Guo B. (2018). Association of periodontitis with rheumatoid arthritis and the effect of non-surgical periodontal treatment on disease activity in patients with rheumatoid arthritis. Med. Sci. Monit. Int. Med. J. Exp. Clin. Res..

[100] Kim J.H., Choi I.A., Lee J.Y., Kim K.H., Kim S., Koo K.T., Kim T.I., Seol Y.J., Ku Y., Rhyu I.C. (2018). Periodontal pathogens and the association between periodontitis and rheumatoid arthritis in korean adults. J. Periodontal Implant. Sci..

[101] Lee J.Y., Choi I.A., Kim J.H., Kim K.H., Lee E.Y., Lee E.B., Lee Y.M., Song Y.W. (2015). Association between anti-porphyromonas gingivalis or anti-alpha-enolase antibody and severity of periodontitis or rheumatoid arthritis (ra) disease activity in ra. BMC Musculoskelet. Disord..

[102] Bizzarro S., Van der Velden U., Loos B.G. (2016). Local disinfection with sodium hypochlorite as adjunct to basic periodontal therapy: A randomized controlled trial. J. Clin. Periodontol..

[103] Bizzarro S., van der Velden U., Teeuw W.J., Gerdes V.E.A., Loos B.G. (2017). Effect of periodontal therapy with systemic antimicrobials on parameters of metabolic syndrome: A randomized clinical trial. J. Clin. Periodontol..

[104] Bialowas K., Radwan-Oczko M., Dus-Ilnicka I., Korman L., Swierkot J. (2020). Periodontal disease and influence of periodontal treatment on disease activity in patients with rheumatoid arthritis and spondyloarthritis. Rheumatol. Int..

[105] He Y., Yang F.Y., Sun E.W. (2018). Neutrophil extracellular traps in autoimmune diseases. Chin. Med. J. Engl..

